# Supplementary material for: Targeting MYC Regulation with Polypurine Reverse Hoogsteen Oligonucleotides
Source: Int J Mol Sci. 2022 Dec 26;24(1):378. doi: 10.3390/ijms24010378 (PMC9820101; doi:10.3390/ijms24010378)
Supplement: Supplementary file 1 [file ijms-24-00378-s001.zip › ijms-2039080-supplementary.pdf]

## Supplementary Files/Figures

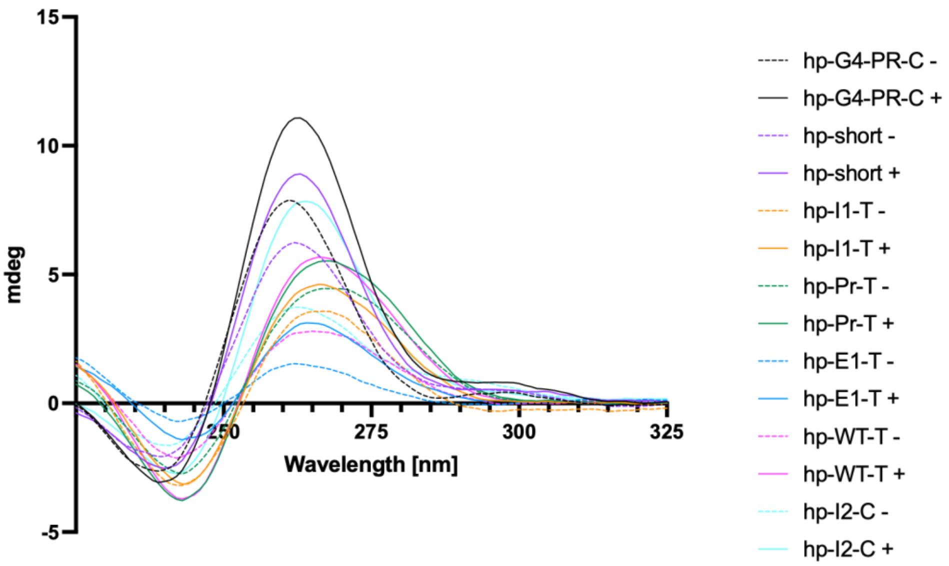


**Figure S1.** G4 and hairpin formation within the PPRH sequences**.** ECD was used to establish the non-canonical DNA formations, if any, formed from the PPRHs in the absence (-, dashed lines) or presence (+, solid lines) of 100 mM KCl. Parallel G4 formation is identified by a maximal Cotton effect in the 262-264 nm range, as identified with the hp-G4-PR-C and Hp-I2-C sequences, while hairpin formation was identified in all other PPRH sequences by a maximal (and broad) Cotton effect at 270 nM.

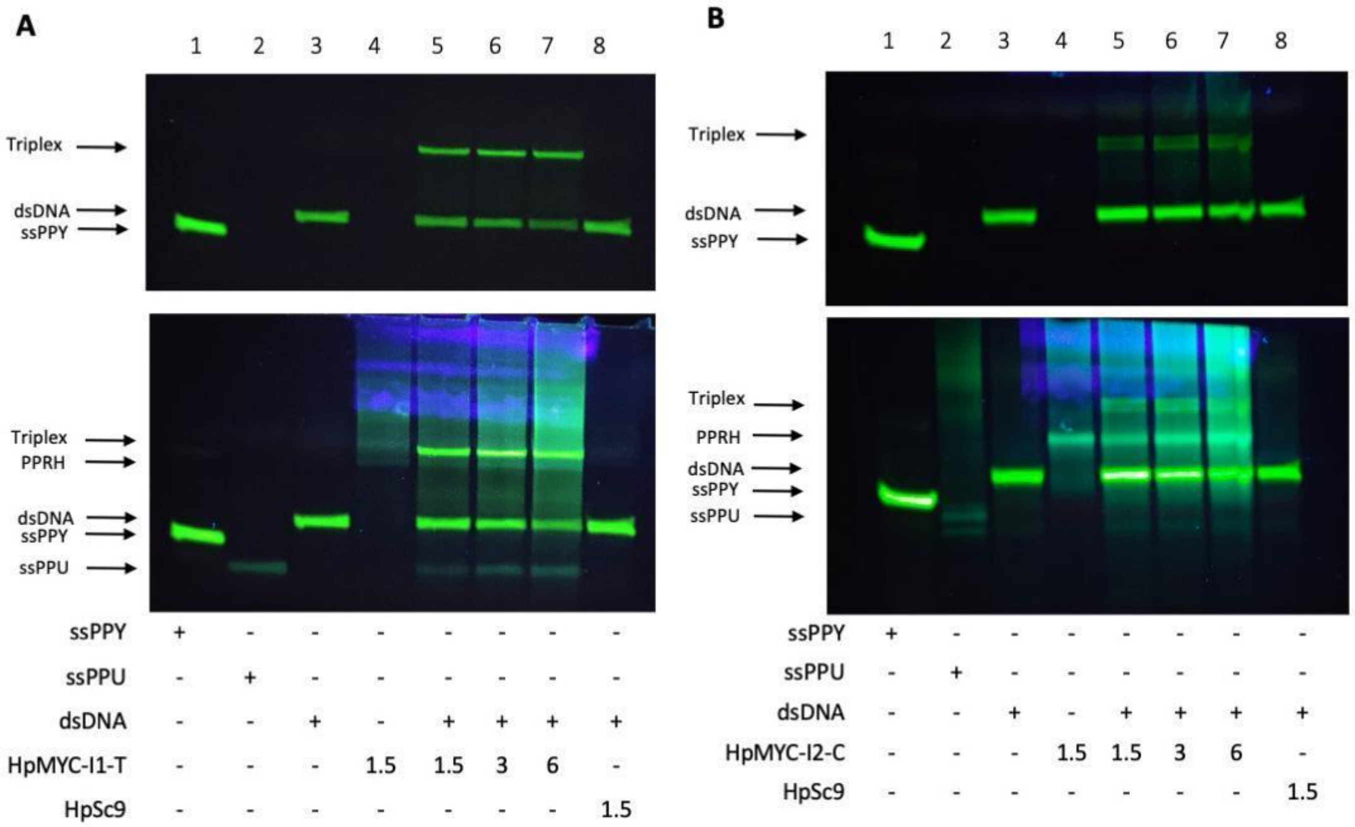

**Figure S2.** Displacement analyses of the Polypurine (ssPPU) strand in intron 1 (A) and Intron 2 (B) probes. (**A**) Displacement analysis of the Polypurine (PPU) strand in Intron 1 probe. (**B**) Displacement analysis of the Polypurine (PPU) strand in Intron 2 probe. Bindings were performed using 1.5 µg of dsDNA labeled with FAM (green) in the polypyrimidine (PPY) strand only, then incubated as described in M&M with increased amounts of their PPRH, HpMYC-I1–T, or HpMYC-I2-C. .
